# Does Resident Rotation Affect the Learning Curve of Active Robotic TKA? A Study of Surgical Efficiency and Radiographic Precision

**DOI:** 10.3390/medicina62030533

**Published:** 2026-03-13

**Authors:** Yong-Beom Park, Jin-Woong Jeon, Seong Hwan Kim, Han-Jun Lee

**Affiliations:** 1 Department of Orthopedic Surgery, Chung-Ang University Gwangmyeong Hospital, Chung-Ang University College of Medicine, Gwangmyeong-si 14353, Republic of Korea; 2 Department of Orthopedic Surgery, Chung-Ang University Hospital, Chung-Ang University College of Medicine, Seoul 06973, Republic of Korea; okjinwoong@gmail.com (J.-W.J.); ksh170177@nate.com (S.H.K.);cauknee@naver.com (H.-J.L.)

**Keywords:** alignment, arthroplasty, learning curve, operative time, robot

## Abstract

*Background and Objectives*: Learning curves robotic arm-assisted total knee arthroplasty (TKA) are well-documented for semi-active systems, but evidence for advanced fully active robotic systems remains scarce. This study aimed to characterize the learning curve for operative time, implant positioning, and lower-limb alignment using a fully active robotic TKA system, specifically accounting for the impact of rotating resident involvement in a tertiary center. *Materials and Methods*: Sixty consecutive primary TKAs were performed using the advanced active robotic system (CUVIS-Joint^®^). The learning curve for operative time was evaluated using cumulative summation (CUSUM) analysis. To identify independent predictors of surgical duration and radiographic precision, a multivariate linear regression model was constructed, including case number, implant type, and resident rotation period as variables. *Results*: CUSUM analysis identified a statistically significant inflection point at the 39th case. Beyond this point, mean operative time decreased approximately 20 min (133.3 ± 13.5 vs. 113.8 ± 7.9 min, *p* < 0.001). Multivariate regression confirmed that case number was the sole independent predictor of operative time (*p* < 0.001). Notably, implant positioning and lower-limb alignment showed no detectable difference across the sequential cases (*p* > 0.05), maintaining high precision from the outset. *Conclusions*: Active robotic TKA demonstrated a learning curve for operative time that stabilized after 39 cases within a clinical setting of rotational resident participation. Radiographic accuracy remained consistent despite these educational requirements, supporting the technical feasibility and reliability of this advanced system for the management of end-stage knee osteoarthritis

## 1. Introduction

Despite the introduction of several technical advancements in total knee arthroplasty (TKA) in terms of patient-specific implants, computer navigation, and enhanced recovery programs [1,2,3,4], patient dissatisfaction remains up to 20% [5,6,7]. Accurate implant positioning, balanced flexion-extension gaps, proper mediolateral ligament balancing, and preservation of the surrounding soft tissue envelope during TKA have been associated with clinical outcomes and implant survivorship [8,9]. Theoretically, robotic technology with enhanced accuracy and reproducibility may help surgeons improve the clinical outcomes, including patient satisfaction [10].

Several robotic systems with varying learning curves have been shown to improve the accuracy of implant positioning and reduce alignment outliers in TKA [11,12,13,14,15]. Robotic systems for TKA are classified as either fully active or semi-active according to the degree of control of the robotic device during surgery. Semi-active robotic arm-assisted TKA has a reported learning curve of 9–30 cases for operation times, depending on the robotic systems [16,17,18,19,20]. Whereas previous active robotic systems have reported high accuracy for preoperative patient-specific plans [3,21,22,23] and have shown a learning curve of 12–19 cases for operation times [24]. The **advanced active robotic system** evaluated in this study allows for intraoperative plan adjustments based on real-time soft tissue evaluation, a feature that distinguishes it from earlier models [25]. However, the learning curve for this advanced fully active robotic system remains unexplored.

The education of residents is crucial for producing highly skilled and well-trained future healthcare providers. Several orthopedic studies have reported the costs, outcomes, and adverse effects of resident participation with conflicting data [26,27]. A recent study on TKA with resident participation reported an increased operative time without jeopardizing short-term patient clinical outcomes, satisfaction, and complications [28]. However, the impact of rotational resident participation on the learning curve of robotic systems for TKA has rarely been investigated.

Therefore, this study aimed to assess the learning curve of a novel advanced active robotic system for primary TKA. specifically characterizing operative time, implant positioning, and lower limb alignment within an academic environment involving rotational resident participation.

## 2. Materials and Methods

This prospective cohort study included the first consecutive patients undergoing primary TKAs with the newly advanced active robotic system (CUVIS Joint^®^, CUREXO, Seoul, Republic of Korea) from July 2020 to February 2022. Apart from semi-active robotic arm-assisted systems, the fully active robotic arm performed bone resection under the guidance and supervision of the surgeon. This study was approved by the Institutional Review Board of Chung-Ang University Hospital (protocol code 2012-011-441, 8 March 2021), informed consent was obtained from all subjects involved in the study. The patients treated between July 2020 and March 2021 received the robotic TKA as part of standard clinical care following the institutional adoption of the system. Prospective data collection and formal study enrollment strictly commenced following the IRB approval on 8 March 2021. All consecutive cases from the initial implementation were included to provide a comprehensive analysis and minimize selection bias.

The open implant platform of the robotic system can accommodate different implants depending on the match between the implant and the patient’s anatomical dimensions. In this study, the Lospa^®^ posterior-stabilized type (CORENTEC Inc., Seoul, Republic of Korea) [29] and Nexgen LPS-flex^®^ posterior-stabilized type (Zimmer Inc., Warsaw, IN, USA) were implanted during robotic TKAs.

### 2.1. Surgical Technique

**Figure 1:**
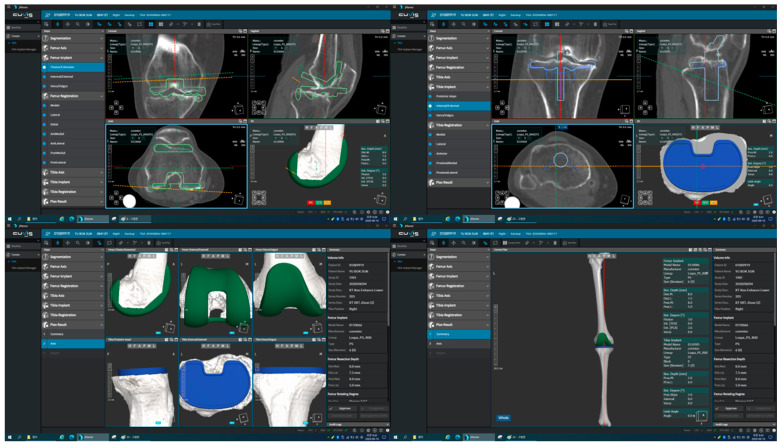
Preoperative planning for positions and alignments of femoral and tibial implants using J-Planner (version 1.01).

**Figure 2:**
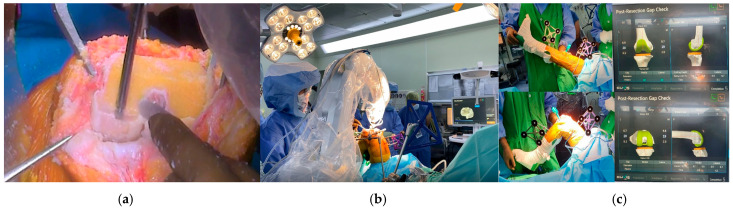
Intra-operative surgical procedures in advanced robotic total knee arthroplasty. (**a**) Bone resection using the active robotic arm with a milling burr, (**b**) Surgeon supervision, (**c**) Real-time feedback for accurate soft tissue balancing.

In the Department of Orthopedic Surgery at our tertiary center, residents change their division every 4 months. Two residents participated in TKA. An experienced surgeon performed all active robotic TKAs using a medial parapatellar approach. Before performing active robotic TKA, the surgeon established a preoperative plan for optimal implant design, size, and positioning based on preoperative computed tomography (CT) images (Figure 1). Following adequate surgical exposure, two trackers (one each on the femur and tibia) were rigidly fixed. After registration of the bony landmarks, the agreement between the operated knee and virtual knee based on CT and registration was evaluated [25]. After confirming the agreement, the active robot and operated knee were rigidly docked using clamps. Bone resection was performed using an active robotic arm with a milling burr according to the preoperative plan or a revised intraoperative plan (Figure 2). During active milling bone resection, the surgeon supervised the process to protect the soft tissue envelope and ensure efficient removal of bone remnants (Figure 2). Trial implants were inserted and soft tissue balancing was assessed and achieved using real-time feedback from the newly advanced active robotic system (Figure 2). Definite implants were inserted using cement, and the patella was non-resurfaced in all patients.

### 2.2. Radiological Measurements

**Figure 3:**
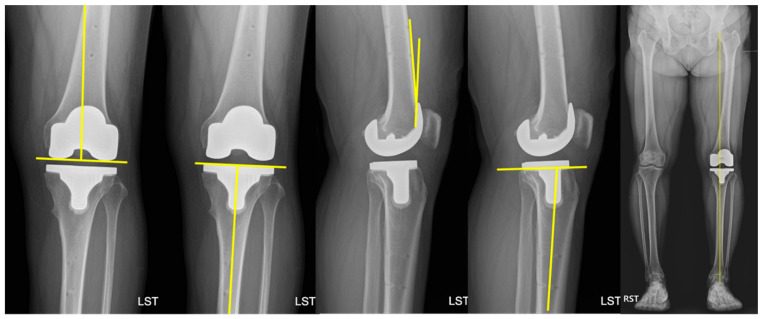
Assessment of component alignment and lower limb alignment. Coronal femoral component angle (α), tibial component angle (β), sagittal femoral component angle (γ), tibial component angle (δ), and hip-knee-ankle angle.

Full-length standing anteroposterior radiographs of the lower extremities were used to evaluate the mechanical hip-knee-ankle (HKA) angle [30,31], which indicates lower limb alignment; varus alignment was set as positive for HKA. The implant positions in the coronal and sagittal planes, including coronal femoral component angle (α), coronal tibial component angle (β), sagittal femoral component angle (γ), and sagittal tibial component angle (δ), were assessed using the knee anterior-posterior and lateral radiographs (Figure 3) [32]. All measurements were performed using a picture archiving and communications system (PACS; General Electric, Chicago, IL, USA) monitor using a mouse point cursor and automated computer calculations; the values were rounded off to two decimal places.

### 2.3. Operative Time

Total operative time was measured as the duration from the tourniquet inflation before skin incision to its depletion after compression dressing application.

### 2.4. Data Analyses

The learning curve for operative time was analyzed using the cumulative summation (CUSUM) method [33,34]. To statistically validate the inflection point of the learning curve, a segmented regression analysis (piecewise regression) was performed. The target operative time for the CUSUM analysis was predefined as 113.8 min, which represents the mean operative time achieved during the proficiency phase (Cases 40–60). This allowed for a more rigorous identification of the structural break in surgical efficiency. The operative time, implant positioning, and lower limb alignment were aggregated into chronological groups of 10 cases. A total of 60 patients were classified into six groups, and comparisons among groups were analyzed using Mann–Whitney or Kruskal–Wallis tests for non-parametric variables, whereas independent *t*-test or one-way ANOVA were used for normally distributed continuous variables.

Furthermore, a multivariate linear regression analysis was performed to identify independent predictors of operative time and radiographic precision. The model included case number (experience), implant type, and the resident rotation period as independent variables to account for potential confounding factors within the institutional environment. The significance level was set at *p* < 0.05. All statistical analyses were performed using the SPSS software ver. 28.0 (SPSS Inc., Chicago, IL, USA).

## 3. Results

A total of 60 consecutive patients were enrolled in the study, with a mean age of 73.6 years. The demographic details are shown in Table 1.

### 3.1. Operative Time

**Figure 4:**
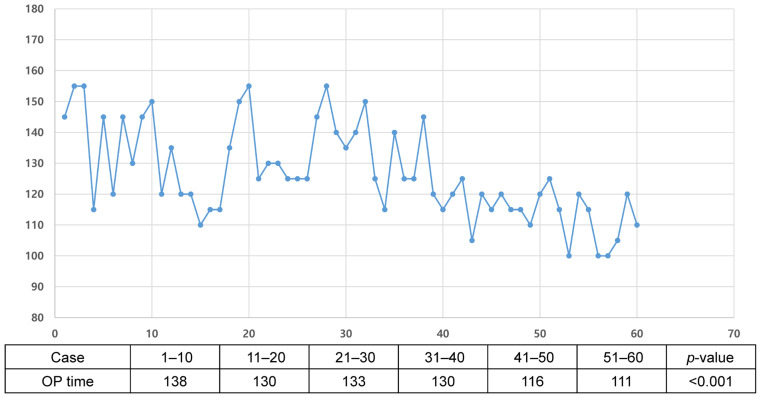
The distribution of operation time for each case.

**Figure 5:**
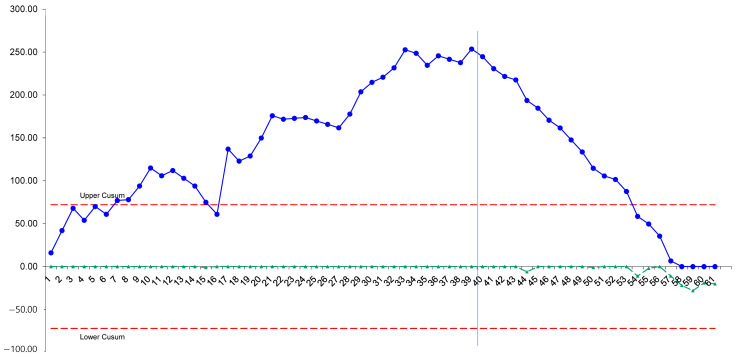
The cumulative summation analysis for operation time.

The active robotic TKA showed a mean operative time of 126.5 min. Based on the comparison of the chronological groups, active robotic TKA was associated with a learning curve of >40 cases for operative time (*** *p* < 0.001) (Figure 4). To confirm this, a segmented regression analysis was performed, which identified a statistically significant structural break in the learning slope at the 39th case (*p* < 0.001). Consistent with this, the inflection point of the CUSUM analysis was at the 39th case from the initial case (Figure 5). After 39 cases, the operative time was significantly decreased by 20 min (133.3 ± 13.5 min from the first case to the 39th case, and 113.8 ± 7.9 min from the 40th case to the 60th case, *** *p* < 0.001).

### 3.2. Lower Limb Alignment and Implant Positioning

Limb alignment showed a mean deviation of 1.6° towards the varus postoperatively compared to the intraoperative plan (HKA angle: 1.6° ± 1.5°). Target lower limb alignment (<± 3° deviation from neutral) was achieved in 95% (57/60) of the TKAs.

The average deviation for the coronal planes of the femoral and tibial implants from the preoperative plan was <1.0° (Table 2). The precision of implant positioning and lower-limb alignment showed no detectable difference across the sequential groups (Table 3).

### 3.3. Factors Affecting Surgical Efficiency and Accuracy

A multivariate linear regression analysis was performed to identify independent predictors of surgical outcomes (Table 4). For operative efficiency, case number was identified as the only significant independent predictor of reduced tourniquet time (beta = −0.72, *p* < 0.001), explaining 58.2% of the variance (R^2^ = 0.582). In contrast, implant type (*p* = 0.412) and the resident rotation period (*p* = 0.285) showed no significant correlation with operative time.

Regarding implant positioning (Alpha, Beta, Gamma, and Delta angles) and lower-limb alignment (Postoperative HKA angle), none of the analyzed variables—case number, implant type, or resident rotation—significantly influenced these radiographic outcomes (all *p* > 0.05).

## 4. Discussion

The most important finding of this study was the learning curve of 39 cases associated with the introduction of a newly advanced fully active robotic system for TKA. Following this learning curve, the operation time decreased by approximately 20 min, within a clinical setting of rotational resident participation. Notably, our multivariate linear regression analysis further strengthens these findings by isolating the impact of cumulative experience from other potential confounders within the institutional environment. While surgical efficiency significantly improved as the chronological case number increased, the transition between resident rotation groups did not statistically influence operative time, lower-limb alignment, or implant positioning (all *p* > 0.05).

The learning curve for the operative time in fully active robotic TKA was 39 cases, which is longer than that in semi-active robotic arm-assisted TKA of 7–30 cases [16,17,18,19,20,35,36,37]. Determining the reason behind the higher number of cases in the learning curve of active robotic systems was challenging. We initially anticipated that the participation of rotational residents might have contributed to this difference. However, the multivariate analysis revealed that resident rotation was not a statistically significant factor. Instead, the extended curve likely reflects the complexity of mastering the fully active autonomous workflow and the deliberate verification process required for bone resection accuracy. Although the bone resection was performed by the surgeon under haptic feedback using semi-active robot arm-assisted systems, active robotic arm resected the bone using a milling burr according to the pre-defined plan, under the supervision of the surgeon [25]. Consequently, the structured workflow and real-time feedback mechanisms of this advanced system may mitigate the variability typically associated with changes in the surgical team.

In this context, the 39-case threshold represents the point at which the entire surgical team reached a ‘steady state’ of efficiency, accounting for the inherent educational demands and staff turnover. Moreover, after the learning phase, the operation time did not change significantly despite the continued participation of rotational residents. During the proficiency phase, involving a surgical team that includes residents becomes increasingly feasible, maintaining both efficiency and radiographic precision.

The change in the operation time of active robotic TKA was similar to that previously reported for semi-active robotic arm-assisted TKA despite differences in case numbers required for achieving proficiency. In this study, the operative time of active robotic TKA decreased by 19.5 min (133.3 min [1st case to 39th case] in the learning phase to 113.8 min [40th case to 60th case] in the proficiency phase). Only three studies on semi-active robot arm-assisted TKA systems reported the operation times in the learning and proficiency phase [12,35,37], with a mean change of 23.9 min. These findings suggest that primary TKAs have a decreased operation time of approximately 20 min, regardless of the type of robotic system, after the learning curve.

Active robotic TKA showed no detectable difference in achieving the planned implant positioning. The multivariate analysis confirmed that radiographic accuracy—including postoperative HKA angle and implant component positions—remained consistently high from the outset and was not significantly affected by cumulative experience, implant type, or resident rotation (all *p* > 0.05). This may have been due to the high accuracy of the advanced robotic system in bone resection in terms of thickness and alignment [25]. The accuracy of the newly advanced robotic system was comparable to that of other robotic TKA systems in previously reported cadaveric studies [13,38,39]. Hence, the high precision of this robotic system supports its feasibility for broad clinical applications.

The strength of this study was the consideration of rotational resident participation, a factor largely unexplored in robotic TKA learning curve literature. While a previous study on conventional TKA reported that resident participation increased operative time without jeopardizing clinical outcomes [28], our results demonstrate that the standardized procedural steps of this advanced robotic system allow for resident education without compromising surgical efficiency or radiographic accuracy once initial proficiency is reached. These findings should help alleviate concerns among surgeons and patients regarding active robotic TKA procedures performed within a teaching hospital environment.

This study had some limitations. First, there was an absence of a control group that underwent conventional jig-based or robot-assisted TKA (semi-active). However, our primary focus was strictly on the learning curve associated with the initial adoption of a newly advanced active robotic system. Secondly, as only one surgeon was included, this study essentially represents a personal adoption curve, which may limit the generalizability of the 39-case learning curve to other surgeons or institutions. Nevertheless, previous studies on the learning curves of semi-active systems have reported relatively uniform results with only slight variations [37,40]. Thus, we anticipated that focusing on the impact of rotational resident participation in a tertiary center would provide more informative data for academic environments. Third, the level of resident training and the specific proportion of operative steps performed were not stratified quantitatively, which may influence the interpretation of the learning curve. However, our multivariate analysis mitigated this limitation by demonstrating that the transition between different resident groups did not statistically influence surgical efficiency or radiographic precision. Finally, the lack of long-term clinical outcomes, such as patient-reported scores or complication rates, limits the assessment of the system’s overall clinical efficacy. Future multi-center studies involving diverse surgical teams and long-term follow-up are necessary to validate these findings further. Additionally, the use of two different implant systems and the consistent practice of patellar non-resurfacing reflect the specific surgical protocols of our institution, which may limit the direct generalizability of these results to centers with different practices.

## 5. Conclusions

Active robotic TKA was associated with a learning curve of 39 cases for operative time within a clinical setting of rotational resident participation in a tertiary center. Multivariate linear regression analysis confirmed that radiographic accuracy in implant positioning and lower-limb alignment remained consistently high from the outset and was not significantly affected by cumulative experience or changes in the surgical team. In conclusion, these findings demonstrate that this advanced active robotic system is a technically feasible and reliable tool for the surgical management of end-stage knee osteoarthritis in academic environments.

## Figures and Tables

**Table 1:** Baseline characteristics.

Characteristics	
Age (years)	73.6 ± 5.0
Body mass index (kg/m^2^)	27.3 ± 3.7
Gender (Women/Men)	55 (91.7%)/5 (8.3%)
Side (right/left)	29 (48.3%)/31 (51.7%)
Hip-knee-ankle angle (°)	Varus 7.7 ± 4.9

**Table 2:** The positions of femoral and tibial components.

Angle	Criteria	Angle	Proportion (n = 60)
α	90° ± 3°	89.1°	59 (98.3%)
β	90° ± 3°	89.3°	58 (96.7%)
Γ	0° ± 5°	2.8°	56(93.3%)
δ	87° ± 3°	85.6°	55(91.7%)

**Table 3:** The comparisons of alignments according to the serial cases.

	1st to 10th	11th to 20th	21st to 30th	31st to 40th	41st to 50th	51st to 60th	*p*-Value
HKA	1.9	1.5	1.7	1.5	1.6	1.4	0.126
α	89.1	89.3	89.0	89.2	89.1	89.0	0.845
β	89.4	89.1	89.3	89.3	89.2	89.5	0.548
γ	3.0	2.7	2.9	2.8	2.7	2.7	0.779
δ	85.5	86.1	85.3	85.6	85.4	85.7	0.128

HKA: Hip–Knee–Ankle.

**Table 4:** Multiple linear regression model accounting for surgical outcomes.

Dependent Variable	Independent Variable	Standardized Coefficient (β)	*p*-Value
Operative Time	Case Number (Experience)	−0.72	<0.001
	Implant Type	0.12	0.412
	Resident Rotation Group	−0.08	0.285
Lower-limb alignment (HKA Angle)	Case Number (Experience)	0.05	0.652
	Implant Type	−0.03	0.774
	Resident Rotation Group	0.04	0.725
Implant Positioning (Component Angles)	Case Number (Experience)	0.04	0.587
	Implant Type	0.02	0.602
	Resident Rotation Group	0.03	0.554

## Data Availability

The datasets used and analyzed during the current study are available from the corresponding author on reasonable request.

## Abbreviations

The following abbreviations are used in this manuscript:

TKA	Total Knee Arthroplasty
CT	Computed Tomography
HKA	Hip–Knee–Ankle
CUSUM	Cumulative Summation
